# Physical fitness indicators and physical activity behind self-determined motivation in Emirati female college students

**DOI:** 10.3389/fspor.2026.1855203

**Published:** 2026-07-07

**Authors:** Arto Gråstén, Balazs Gabor, Gehan Handouk, Jamal Alnuaimi

**Affiliations:** Department of Physical Education, College of Education, United Arab Emirates University, Al Ain, Abu Dhabi, United Arab Emirates

**Keywords:** autonomous motivation, body composition, controlled motivation, United Arab Emirates, young women

## Abstract

**Background:**

Physical inactivity is a public health concern, especially among young Middle Eastern women. Individuals with higher levels of physical fitness are more likely to engage in regular moderate-to-vigorous physical activity (MVPA), as lower physical effort may reduce discomfort during activities and enhance motivation to remain active.

**Methods:**

This follow-up study examined physical fitness indicators and MVPA related to self-determined motivational regulation among Emirati female college students. A total of 254 participants (aged 18–24 years) completed questionnaires and objective assessments of MVPA, body composition (fat and muscle mass), and muscular strength (handgrip). Motivational regulations were assessed at baseline (T0) and eight weeks later (T1), while laboratory measures and MVPA were collected at baseline (T0). A path model, including MVPA, fat and muscle mass, and handgrip strength as independent variables and motivational regulations as dependent variables, was tested.

**Results:**

Findings showed that handgrip strength was positively associated with intrinsic, integrated, and identified regulation, while higher MVPA was positively associated with identified regulation and negatively associated with non-regulation. Higher fat mass and lower muscle mass were associated with greater non-regulation. As expected, motivational regulations remained stable over eight weeks, indicating constant self-assessments.

**Discussion:**

Promoting engagement in muscle-strengthening activities and MVPA among Emirati female college students may foster more autonomous motivation for physical activity and support long-term health outcomes.

## Introduction

Regular physical activity is essential for physical and mental health (1, 2), yet participation among young women in the Middle East remains low (3, 4). Higher levels of physical fitness enable individuals to sustain greater physical activity intensity and longer durations with less fatigue (5). When activities are easier to manage, individuals are more likely to persist, and this persistence links to motivation, potentially enhancing long-term physical activity participation (6). Self-Determination Theory (SDT) provides a useful framework for understanding physical activity motivation (7, 8). However, most SDT-based evidence derives from Western contexts (9, 10), limiting generalisability to regions where cultural (e.g., societal norms regarding participation in exercise and sport), social (e.g., family and peer support for physical activity), and environmental factors (e.g., access to recreational facilities and weather conditions) shape physical activity behaviour differently (11, 12). While motivation is typically treated as a determinant of physical activity, it is increasingly recognised as a complex, multidimensional construct that behavioural and physical characteristics may also shape (13). Addressing these gaps, the present study examined body composition, moderate-to-vigorous physical activity (MVPA), and muscular strength as antecedents of SDT-based motivational regulations among Emirati female college students.

According to SDT, motivation exists along a continuum ranging from amotivation (a lack of motivation) to extrinsic motivation (engaging in an activity for external rewards or outcomes) and intrinsic motivation (engaging in an activity for interest or enjoyment) (7, 8). The basic psychological needs for autonomy (a sense of choice), competence (feeling capable of achieving desired outcomes), and social relatedness (feeling connected to others) act as antecedents to motivation, facilitating their development and internalization (7, 8). In addition, motivational regulations represent the specific ways in which behaviour is regulated and reflect the source of motivation. Thus, motivational regulations are the specific constructs that vary in the extent to which behaviour is self-determined. Non-regulation reflects a lack of intention or perceived value in the activity. This is followed by more controlled forms of regulation, including external regulation, driven by external rewards or pressures, and introjected regulation, which is influenced by internal pressures such as guilt or obligation. As motivation becomes more internalised, it shifts toward more autonomous forms. These include identified regulation, in which physical activity is personally valued, and integrated regulation, in which it is aligned with broader goals and an individual's identity. The most self-determined form is intrinsic regulation, where activity is undertaken because it is interesting and enjoyable. Considering the complexity of motivation (13) and its associations with behavioural (14) and physical characteristics (15, 16), motivational regulations were examined in the current study as the primary constructs of motivation, rather than the psychological needs underlying their evolvement. Examining these regulations may be particularly relevant in populations with low levels of physical activity, such as young adults in the Middle East (3, 4).

Previous studies have shown that only approximately 25% of young people in the Middle East meet current MVPA recommendations, with participation particularly low among females (3, 4). Motivational regulations are recognised as important determinants of physical activity behaviour, with more self-determined forms of motivation consistently associated with better adherence to physical activity (10, 17), higher levels of MVPA (9, 10, 13, 18–20), and greater intention to be active (21). In contrast, non-regulation and controlled forms of motivation are generally associated with lower levels of physical activity among children and adolescents (22, 23). Despite this evidence, research has largely conceptualised physical activity as an outcome of motivation rather than a potential antecedent for motivational regulation (10, 13, 20). Although physical activity has been linked to academic motivation (24, 25), physical self-efficacy (26), and psychological well-being (27), its role in shaping SDT-based motivational regulations remains underexplored. This represents an important theoretical gap, as regular engagement in MVPA may contribute to the development of more self-determined forms of motivation through positive experiences of competence, achievement, and behavioural success (7, 8). Examining physical activity as an antecedent of motivational regulation, therefore, extends SDT by investigating a potentially reciprocal relationship between MVPA engagement and motivation among university students. In addition to behavioural factors such as MVPA engagement, other physical characteristics may also contribute to the development of motivational regulations.

Body composition may represent an important physical antecedent of motivational regulation, as it can influence individuals' experiences during physical activity through their perceptions of physical competence (7, 8). Previous research has shown that higher body mass index (BMI) is associated with lower self-determined motivation and higher non-regulation (28, 29), potentially due to negative body image, reduced perceived competence, or physical discomfort during activity. Consistent with these findings, a study conducted in Saudi Arabia reported that students across all BMI categories exhibited intrinsic motivation. However, normal-weight and more physically active students demonstrated stronger autonomous forms of motivation (intrinsic and identified regulation), whereas obese students reported higher levels of extrinsic motivation and non-regulation (30). These findings suggest that body composition may influence motivation for physical activity by shaping individuals' physical experiences and self-perceptions. Given the established associations between body composition and motivational regulation, further investigation is warranted to understand better how physical characteristics contribute to more autonomous forms of motivation. However, body composition is not the only physical attribute that may shape motivational experiences.

Muscular strength may also be relevant to motivational regulation, although this relationship remains largely unexplored. Muscular fitness is a well-established determinant of favourable health outcomes and reduced risk of chronic disease (31–33). In particular, handgrip strength has been recognised as a reliable indicator of overall health, functional capacity, and physical fitness (31, 32). As a simple marker of physical capability, greater muscular strength, together with higher muscle mass, may also reflect enhanced perceptions of physical competence, which SDT identifies as an important antecedent for more self-determined motivation (7, 8). While other measures of muscular strength could be equally relevant, handgrip strength is a valid, practical, and extensively used indicator of overall physical fitness and strength (31, 32). No empirical SDT-based studies have directly examined the relationship between muscle strength and motivational regulation among college students. Existing research has independently linked muscular fitness to health outcomes (31, 32) and to lower risks of chronic illnesses (33), but its association with motivational regulation remains unclear. This shortcoming highlights the need for a study that integrates physiological indicators with SDT-based motivational frameworks. Addressing this gap requires consideration of physical, behavioural, and motivational characteristics.

The current literature review revealed that physical activity and physical fitness are associated with a range of psychological and health-related outcomes. MVPA, body composition indicators, and muscular strength are linked to MVPA engagement and well-being (13–16), while physical activity predicts academic motivation (24, 25), physical self-efficacy (26), and psychological well-being (27). However, little is known about whether physical activity and physical fitness act as antecedents of SDT-based motivational regulation. Although SDT identifies motivation as a driver of engagement in activities (7, 8), behavioural and physical characteristics may also shape motivational regulation through physical competence and experiences. Furthermore, existing evidence on motivation for physical activity is largely based on cross-sectional and intervention studies. More self-determined motivation predicts sustained engagement in physical activity (30, 34). However, less is known about how motivational regulations function under real-world everyday conditions without manipulation (35). This limits our understanding of the extent to which motivational processes observed in research settings translate to daily physical activity behaviour. This is particularly evident in Middle Eastern contexts, where SDT-based research remains limited (36, 37), especially among female university students who may face sociocultural barriers to participation in physical activity (38–40). Accordingly, examining the relationships between physical activity, physical fitness, and motivational regulation among Emirati female college students addresses important gaps in the current literature.

**Figure 1:**
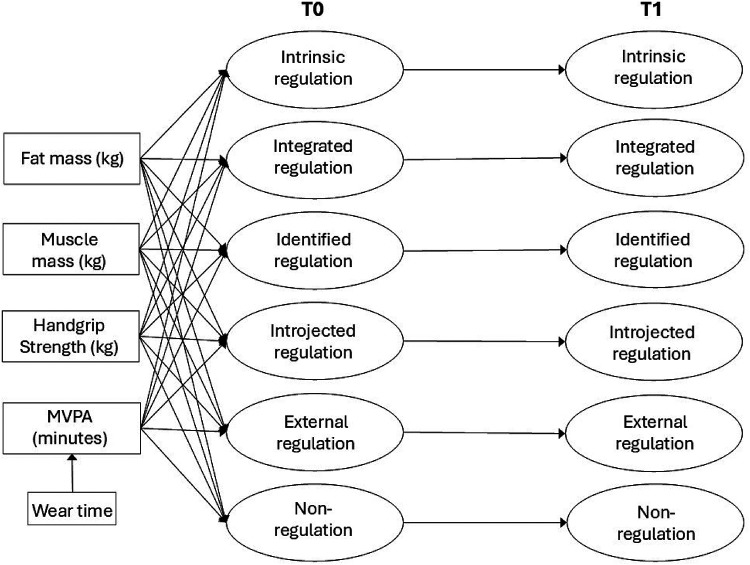
The theorized model of the study. Oval refers to latent and rectangular to observed variables.

The aims of this study were: 1) to investigate the antecedents of motivational regulations by exploring the roles of MVPA, fat mass, muscle mass, and handgrip strength associated with various forms of motivation in Emirati female college students, and 2) to examine the stability and variance of motivation regulation over two time points (Figure 1). Based on previously established associations between motivation and health outcomes, it was expected that higher MVPA (10, 13, 20), greater muscle mass (28–32), and stronger handgrip strength would be positively associated with greater self-determined motivational regulation. In contrast, lower MVPA, handgrip strength, and muscle mass, as well as higher fat mass, would predict lower autonomous motivation and higher non-regulation.

## Methods

### Participants

Participants were recruited from higher education institutions using public announcements and direct outreach. Eligibility criteria included being female, currently enrolled in college, and willing to participate voluntarily. The final sample comprised 254 female college students from the United Arab Emirates (UAE), aged 18 to 24 years (M = 20.67, SD = 1.89). This group represents a specific demographic, young adult women in higher education, allowing for focused analysis. Recruitment was challenging due to cultural norms and regulatory constraints often present in Middle Eastern contexts. Despite these obstacles, targeting female college students yielded valuable insights into motivational regulations of physical activity in this population. However, the findings should be interpreted with caution, as they may not be generalizable to broader or different populations, especially in Western contexts.

### Procedure

Before participating, all students provided written informed consent. Data collection took place between February and November 2023 and included questionnaires, laboratory assessments, and physical activity monitoring. Background information, such as gender and date of birth, was collected through an online form accessible via a link emailed to each participant. Motivational regulation was assessed at baseline (T0) and eight weeks later (T1). An eight-week interval was considered sufficient to evaluate measurement invariance and temporal stability, as substantial changes in motivational regulation strategies were not expected even over longer periods (41). This timeframe also helped maximize participant retention and data completeness. Laboratory-based measures and physical activity were assessed at baseline (T0), as they were considered relatively stable over the study period. Repeated measures were unlikely to provide additional information and would have increased participant burden. Students were divided into six groups to ensure the timely completion of laboratory measurements. Each group was supervised by one researcher, with one to two female research assistants aiding with the assessments. Physical activity data were recorded both on and off campus, including Fridays, which hold cultural and religious significance as a day of worship. This scheduling ensured a thorough capture of activity patterns across different contexts. The study protocol received ethical approval from the researchers’ institutional ethics committee.

### Measures

Motivational regulation was assessed using the modified version of the Sport Motivation Scale II (SMS II) (42), in which references to “sport” were replaced with “physical activities.” This scale comprised 18 items, assessed six types of motivational regulation (three items for each), such as Intrinsic (e.g., it is exciting to learn how I can improve), Integrated (e.g., practicing physical activities reflects the essence of who I am), Identified (e.g., I found it is an excellent way to develop aspects of myself that I value), Introjected (e.g., I would not feel worthwhile if I did not), External (e.g., I think others would disapprove of me if I did not), and Non-regulation (e.g., I have the impression that I am incapable of succeeding in physical activities). Participants responded to the item stem: “Why do you participate in physical activities?” using a five-point Likert scale, where 1 = strongly disagree and 5 = strongly agree. The SMS II is a valid and reliable instrument for assessing motivational regulation in sports-related contexts (42).

Handgrip strength was assessed using a Lafayette hand dynamometer (0 to 100 kgs). Students were measured in the physical education laboratory. Before measurements, the dynamometer's accuracy was pre-tested with a 4 kg calibration weight. Participants sat with their feet on the ground and their tested hand at their side, with the elbow bent at a 90-degree angle. They squeezed the dynamometer as hard as possible for three seconds without moving the arm, ensuring it did not touch any body part or object. The right hand was measured first, followed by the left. After five minutes, the protocol was repeated for both hands. Handgrip strength was the total of right and left strength (kg). Data included two measurements: test and retest. Norms for combined grip strength scores were applied (43).

Hand size was measured as the length and width of the palm of the right hand (44). A hand-shaped figure with a scale was placed on the table. The students were asked to place their hands on the figure, and a trained research assistant verified the measurements. Hand length (cm) was determined as the distance from the midpoint of the distal wrist crease to the most distal point of the middle finger. Hand width (cm) was measured from the lateral surface of the metacarpal to its medial surface. In the data, hand size scores were calculated as the product of length and width (cm^2^). Hand size was assessed because a larger hand was expected to be associated with greater handgrip strength (44).

Moderate-to-vigorous physical activity was measured using Actigraph GT3X + accelerometers (Pensacola, FL, USA). Students wore these devices for four consecutive days, specifically two on weekdays and two on weekends. They were instructed to keep the monitors on during waking hours (6 am to 10 pm), excluding swimming and other water-related activities. The devices could be worn over or under clothing and did not need direct skin contact. Active time was operationalized as device wear time, whereas sedentary time corresponded to periods of non-wear. Overall, students engaged in physical activity of any intensity for an average of 36% of their waking hours (range: 3%–82%). The weekly time dedicated to physical activity across intensity levels was calculated from the minutes spent at each level and the total wear time. The devices recorded data in raw mode at a sampling frequency of 30 Hz and compiled 1,800 accumulated samples for each active axis every 60 s. Thresholds for adults aged 18 and over were used to define moderate (2020-5998 cpm) and vigorous (5,999 + cpm) intensity activities (45).

Body composition, including estimated fat mass and muscle mass, was measured using a Tanita BC-420 MA (Arlington Heights, IL, USA) body composition analyser. The equipment estimates scores using bioelectrical impedance analysis. The standard body type was selected for measurement. During the measurement, the students wore traditional abayas and were barefoot, and a 1 kg weight of cloth was used. Before stepping onto the equipment, their height (cm) was measured using the Seca 713 height-measuring scale. The students were asked to avoid excessive physical activity for 12 h before the measurement, excessive eating and drinking the day before, and any eating and drinking for three hours before the measurement.

### Data analysis

Descriptive statistics, including means, standard deviations, and bivariate correlations, were first examined to characterize the data. Before the main analysis, data were graphically inspected and screened using the Missing Completely at Random (MCAR) (46) test for missing values. Internal consistency of subscales was assessed using Cronbach's alpha. Values above.60 indicate acceptable reliability, values between.60 and.80 indicate moderate reliability, and values below.60 indicate low reliability (47). Lower Cronbach's alpha values may be expected in scales with a small number of items, as the coefficient is sensitive to scale length and tends to increase with more items (48). Confirmatory factor analysis was used to test the hypothesized factor structure of the SMS-II (42) across time points.

Measurement invariance of motivational regulations over two time points was assessed to ensure the constructs were comparable (49). Means and variances in motivational regulations over time were tested using Wald's tests for equality of parameters (50). To address the research questions, a path analysis was conducted with motivational regulations as dependent variables, measured at two time points separated by eight weeks. MVPA, handgrip strength, fat mass, and muscle mass were included as predictors of motivational regulations. Wear time was included as a covariate for MVPA, and hand size was included as a covariate for handgrip strength.

Model fit was evaluated using the chi-square test (*χ*^2^) to assess overall goodness-of-fit, with a non-significant *p*-value indicating an acceptable correspondence between the observed and theoretical distributions. Additional fit indices included the root mean square error of approximation (RMSEA), standardized root mean square residual (SRMR), comparative fit index (CFI), and Tucker–Lewis index (TLI). Values of approximately.06 for RMSEA,.08 for SRMR, and.95 or higher for CFI and TLI indicated acceptable model fit (51). Preliminary analyses were performed using SPSS version 31.0 and structural modelling using Mplus version 8.11.

## Results

### Preliminary analyses

A graphical inspection of the data indicated that the variables were approximately normally distributed, and no significant outliers attributable to data entry or coding errors were identified. The data matrix contained 5.7% missing values (239 out of 4,128 observations), primarily due to incomplete responses on selected measures. The MCAR test was non-significant [*χ*^2^(57) = 60.79, *p* = .341], indicating that the pattern of missingness did not differ from randomness. This result supports the assumption that the missing data were missing completely at random (MCAR). Under MCAR conditions, full-information maximum likelihood (default setting) estimation utilizing all available data to estimate model parameters without imputing missing values has been shown to yield unbiased parameter estimates and standard errors.

Confirmatory factor analysis supported the hypothesized factor structure of the SMS-II scale at T0 and T1. At T0, model fit indices were acceptable [*χ*^2^(98) = 211.56, *p* < .001, RMSEA = .071, 90% CI [.06,.08], CFI = .926, TLI = .897, SRMR = .077], indicating an adequate model fit for the data in the proposed model. Model fit improved at T1 [*χ*^2^(83) = 105.20, *p* = .051, RMSEA = .053, 90% CI [.00,.08], CFI = .966, TLI = .950, SRMR = .059], reflecting good overall fit.

Measurement invariance across two time points was supported for all motivation regulation variables, with configural (factor structure) and metric (factor loadings) invariance established, indicating stable construct representation and comparable item–factor relations over time (Table 1). Scalar (mean level) invariance was also supported for most variables. However, it was not fully met for introjected regulation and non-regulation, indicating that latent means for these variables varied across measurements.

**Table 1:** Measurement invariance testing over two time points.

Variable	Model comparison	X^2^	df	p	Meets invariance criteria
Intrinsic regulation	Metric/Configural	2.22	2	.328	Yes
	Scalar/Configural	2.37	4	.666	Yes
	Scalar/Metric	.15	2	.926	Yes
Integrated regulation	Metric/Configural	.55	2	.758	Yes
	Scalar/Configural	4.82	4	.305	Yes
	Scalar/Metric	4.27	2	.118	Yes
Identified regulation	Metric/Configural	6.01	2	.050	Yes
	Scalar/Configural	8.87	4	.064	Yes
	Scalar/Metric	2.85	2	.240	Yes
Introjected regulation	Metric/Configural	.14	2	.928	Yes
	Scalar/Configural	8.22	4	.083	Yes
	Scalar/Metric	8.07	2	.017*	No
External regulation	Metric/Configural	3.10	2	.211	Yes
	Scalar/Configural	4.75	4	.313	Yes
	Scalar/Metric	1.64	2	.438	Yes
Non-regulation	Metric/Configural	1.16	2	.559	Yes
	Scalar/Configural	12.57	4	.013*	No
	Scalar/Metric	11.41	2	.003**	No

**p < .01, *p < .05.

Internal consistency, examined using Cronbach's alphas, was generally acceptable across time points for most subscales (Table 2), indicating good reliability for intrinsic and identified regulation, acceptable for integrated and external regulation, and marginal reliability for non-regulation and introjected regulation. Despite lower but acceptable reliability estimates for the introjected subscale, converging evidence from reliability estimates, confirmatory factor analysis, and measurement invariance supported the robustness and stability of the SMS-II scale structure across time points.

**Table 2:** Descriptive statistics of the study variables.

Variable	N	Min	Max	Mean	SD	*α*
Intrinsic regulation T0	254	1	5	3.80	.79	.80
Intrinsic regulation T1	239	1	5	3.37	.56	.75 (.85)
Integrated regulation T0	254	1	5	3.19	.74	.65
Integrated regulation T1	239	1	5	3.04	.52	.69 (.73)
Identified regulation T0	254	1	5	3.79	.83	.80
Identified regulation T1	239	1	5	3.40	.57	.80 (.85)
Introjected regulation T0	254	1	5	3.22	.69	.60
Introjected regulation T1	239	1	5	2.91	.44	.60 (.60)
External regulation T0	254	1	5	2.02	.83	.73
External regulation T1	239	1	5	2.46	.63	.71 (.83)
Non-regulation T0	254	1	5	2.65	.84	.64
Non-regulation T1	239	1	5	2.63	.65	.61 (.81)
Fat mass T0 (kg)	254	4.60	61.80	19.28	11.98	-
Muscle mass T0 (kg)	254	34.10	57.10	41.07	4.62	-
Handgrip strength T0 (kg)	254	10.25	32.50	20.91	4.35	-
Hand length (cm)	237	10.00	19.00	16.33	1.06	-
Hand width (cm)	237	6.00	13.00	7.48	1.23	-
MVPA T0 (minutes)	253	.00	498.75	89.17	76.50	-
Wear time (minutes)	254	97.92	789.12	361.23	133.04	-

α = Cronbach's alpha for six items (T0 + T1) in the parentheses.

### Descriptive statistics

Descriptive statistics, including sample sizes, ranges, means, and standard deviations, are reported in Table 2, and correlation coefficients between variables are presented in Table 3. Across time points, motivational regulation scores were generally moderate, with intrinsic and identified regulation exhibiting the highest mean values and external regulation the lowest. Means and variances of intrinsic, integrated, identified, introjected, external, and non-regulation did not differ significantly between T0 and T1 (*p* < .05), indicating consistent self-assessment of motivational regulation across measurements.

**Table 3:** Pearson's correlation coefficients between study variables.

Variable	1	2	3	4	5	6	7	8	9	10	11	12	13	14	15	16
Intrinsic regulation T0	1	.42***	.59***	.19	.71***	.29*	.35***	.02	-.14	-.23	.22**	-.23	-.03	.02	.14*	.11
Intrinsic regulation T1		1	.21	.61***	.29*	.69***	.17	.37**	-.16	-.05	.02	.00	.10	.01	.17	.20
Integrated regulation T0			1	.18	.66***	.20	.55***	-.07	.30***	-.11	.05	-.07	-.14	-.07	-.00	-.02
Integrated regulation T1				1	.25*	.74***	.32**	.48***	.04	.21	.20	.27*	.23	.14	.29*	.14
Identified regulation T0					1	.38**	.51***	.25*	.01	-.21	-.15*	-.07	-.05	.05	.10	.17*
Identified regulation T1						1	.28*	.49***	-.10	-.05	.08	.16	.11	-.03	.13	.09
Introjected regulation T0							1	.30*	.38***	-.02	.31***	.10	-.04	-.07	-.06	-.02
Introjected regulation T1								1	-.04	.14	.16	.40***	.18	.12	.03	.07
External regulation T0									1	.47***	.50***	.34**	.05	-.04	-.05	-.15*
External regulation T1										1	.36**	.60***	.09	.02	.11	-.20
Non-regulation T0											1	.57***	.10	.02	-.06	-.23**
Non-regulation T1												1	.21	.18	.18	-.05
Fat mass T0 (kg)													1	.87***	.37***	.05
Muscle mass T0 (kg)														1	.40***	.12
Handgrip strength T0 (kg)															1	-.01
MVPA T0 (minutes)																1

***p < .001, **p < .01, *p < .05.

Correlation coefficients ranged from small to large. Corresponding regulation constructs across time points were moderately to strongly correlated. Autonomous forms of regulation were positively associated with one another, whereas associations between autonomous regulation and non-regulation or external regulation were generally weak to moderate and negative. Fat mass and muscle mass were positively correlated, and both variables showed moderate positive associations with handgrip strength. MVPA exhibited weak associations with most motivational regulations and other physical variables.

### Associations between motivational regulations at T0 and T1

**Figure 2:**
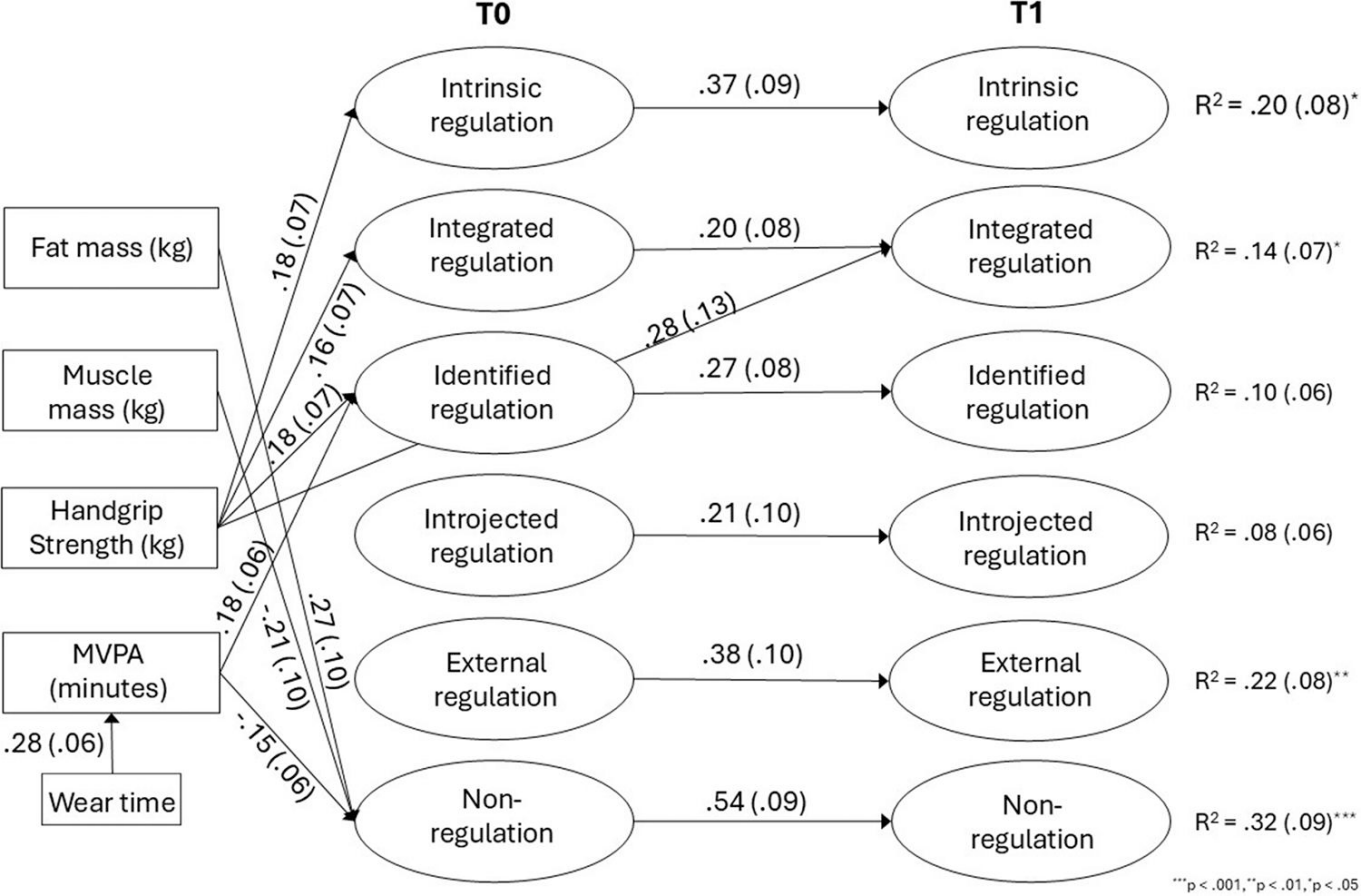
Standardized parameter estimates for motivational regulations and physical fitness indicators at T0 and T1.

To test the associations between motivational regulations, a path analysis was conducted with motivational regulations as dependent variables at two time points, T0 and T1, eight weeks apart. Fat mass, muscle mass, handgrip strength, and MVPA were added in the model as independent variables. Model fit indices indicated acceptable fit to the data [*χ*^2^(45) = 57.29, *p* = .103, RMSEA = .033, 90% CI [.00,.06], CFI = .99, TLI = .96, SRMR = .075]. Hand size was not significantly associated with hand grip strength (*β* = .04, SE = .06, *p* = .511) and was therefore excluded from the final model. Standardized parameter estimates are presented in Figure 2.

At T0, handgrip strength was positively associated with more autonomous forms of regulation, specifically intrinsic, integrated, and identified regulation. MVPA was positively associated with identified regulation and showed a negative association with non-regulation. Neither fat mass nor muscle mass was associated with autonomous motivation outcomes. Fat mass was associated with higher non-regulation, whereas muscle mass was associated with lower non-regulation. No significant associations were observed for introjected or external regulation at T0.

At T1, motivation constructs showed temporal stability, with each T0 regulation corresponding to its T1 outcome. Beyond baseline associations, grip strength was associated with integrated regulation at follow-up, whereas fat mass, muscle mass, and MVPA were not associated with other motivation outcomes. Accelerometer wear time was associated with MVPA, indicating appropriate adjustment for device wear. Squared multiple correlations indicated that the model accounted for a significant proportion of the variance in intrinsic motivation (20%), integrated regulation (14%), external regulation (22%), and non-regulation (32%) at T1.

## Discussion

The present study examined body composition, MVPA, and muscular strength as antecedents of SDT-based motivational regulation among Middle Eastern female college students, thereby evidencing the role of physical fitness indicators in motivational regulation. The main findings were: (1) handgrip strength was positively associated with more autonomous forms of motivational regulation, (2) higher fat mass and lower muscle mass were associated with greater non-regulation, (3) MVPA was positively associated with identified regulation and negatively associated with non-regulation, and (4) stable motivational regulations indicated consistent self-assessment of motivational regulation across measurements.

The finding that higher handgrip strength was associated with more autonomous forms of motivational regulation (intrinsic, integrated, and identified) provides empirical support for the proposition that physical fitness indicators are not merely outcomes of motivation but are meaningfully linked to motivational regulation itself (13). While previous SDT-based research has primarily conceptualised motivation as a predictor of physical activity behaviour (9, 10), the present results extend this framework by demonstrating that muscular strength, a well-established indicator of physical health (31, 32), is associated with more self-determined motivational orientations. This aligns with SDT-consistent evidence showing that experiences associated with physical capability and functional capacity are linked to greater engagement and adherence (17, 18). Importantly, this study is among the first to empirically link muscular strength to SDT-based motivational regulation in college students, thereby expanding SDT's applicability to include objective fitness indicators as correlates of motivational quality in an underrepresented Middle Eastern female population.

The observed association between higher fat mass, lower muscle mass, and greater non-regulation is consistent with existing evidence linking adverse body composition to lower self-determined motivation and higher non-regulation (28, 29). A prior study has shown that individuals with higher BMI exhibit higher levels of non-regulation and controlled regulation, particularly among university students in Middle Eastern contexts (30). The present findings extend this evidence by demonstrating that body composition indicators independently relate to non-regulation, reinforcing the relevance of physical characteristics in associating motivational regulation. Body composition may help explain these findings within the SDT framework. Individuals with less favourable body composition profiles may experience lower perceived physical competence or reduced confidence in their ability to engage successfully in physical activity (52, 53). Additionally, higher BMI may be associated with less positive experiences during physical activity. Together, these experiences may undermine self-determined motivation for MVPA by contributing to feelings of a lack of intention to be active. Within SDT-informed research, non-regulation has been associated with disengagement and low intention to be active (22, 23), and the current results suggest that unfavorable body composition profiles may be linked to motivational disengagement among Emirati female college students. Although the cross-sectional nature of these associations precludes causal inference, the findings are consistent with a reciprocal relationship in which adverse body composition and motivational disengagement reinforce one another over time. These findings underscore the importance of considering body composition as a relevant correlate of non-regulation within SDT-based models of physical activity motivation.

The positive association between greater MVPA and identified regulation, alongside the negative association with non-regulation, is consistent with a substantial body of SDT-based research linking higher levels of physical activity to more self-determined motivational regulation (10, 13, 20). Identified regulation reflects valuing physical activity for its personal importance, and its association with MVPA aligns with evidence that individuals are more likely to engage in sustained activity when they perceive it as meaningful (21). Conversely, the inverse association between MVPA and non-regulation supports previous findings that non-self-determined motivation is associated with lower activity levels (22, 23). Importantly, while prior research has largely examined motivation as a determinant of MVPA, the present findings reinforce emerging perspectives that behavioural engagement itself is closely linked to motivational regulation (13), particularly in everyday, non-intervention contexts.

The absence of significant changes in motivational regulation across time points suggests consistent self-assessment of motivational regulation across measurements in this sample. This finding should be interpreted with caution, as the follow-up period was relatively short (8 weeks) and no intervention or major contextual change was introduced during this interval. However, this finding aligns with prior research indicating that motivational orientations can remain stable in the absence of structured interventions (35). No significant changes in the variances of motivational regulations were found, indicating stable individual-level variability over time. Given the predominance of cross-sectional SDT-based research in Middle Eastern populations (36, 37), these findings extend the literature by demonstrating that motivational regulation may be a relatively stable construct and that change is rare under non-intervention conditions. This highlights the value of non-intervention design for capturing the stability of motivation among Emirati female college students, an underrepresented population facing sociocultural constraints on participation in physical activity (38–40).

A key strength of this study is its two-time-point design, which allowed examination of the stability of motivational regulation rather than relying solely on one-time-point cross-sectional associations. The use of objective measures of MVPA, body composition, and muscular strength strengthens the validity of the findings and reduces bias associated with self-reported physical fitness data. In addition, the focus on Emirati female college students addresses a major gap in SDT and physical activity research by providing evidence from an underrepresented population, thereby enhancing the contextual relevance and generalisability of SDT-based findings beyond Western settings. However, the study has limitations that should be acknowledged. The measurement period was relatively short, limiting the detection of longer-term changes in motivational regulation. In addition, although a two-time point protocol was included, the design does not permit causal or cause-and-effect interpretations regarding motivational regulation indicators. Laboratory-based measures and habitual physical activity were assessed only at baseline due to resource constraints, limiting the evaluation of changes in these variables over time. In addition, body composition was assessed using a Tanita bioelectrical impedance scale, which is subject to variability due to factors such as hydration status and menstrual cycle phase. While the accuracy of body composition estimates may be limited, using a consistent measurement protocol across all participants reduces the likelihood of bias in within-sample associations. However, caution is warranted when comparing results across different samples or studies. As a result, the findings should be interpreted as associative rather than causal, and future studies using longer follow-up periods and experimental designs are needed to clarify directional pathways. In addition, future studies could examine whether unmeasured factors, such as body image or concerns about social evaluation, contribute to these established associations and help explain the underlying mechanisms.

## Conclusions

This study provides evidence that physical fitness indicators are meaningfully associated with motivational regulation for MVPA among Emirati female college students. Greater handgrip strength was consistently associated with more autonomous forms of motivation, while higher MVPA was associated with identified regulation and lower non-regulation. In contrast, higher fat mass and lower muscle mass were associated with greater non-regulation, underscoring the relevance of body composition to motivational disengagement. Together, these findings demonstrate that physical fitness indicators and MVPA are differentially associated with motivational regulation in everyday contexts. While causal relationships cannot be established, the results indicate that strength-focused and competence-supportive activities (e.g., supervised strength training, skill development, and goal progression) may be valuable components of university physical activity programs for young women.

In addition, motivational regulation remained stable over time, with no significant changes observed in any form of regulation. This pattern indicates that, in the absence of intervention effects, autonomous and controlled motivations remain relatively stable over short periods under natural conditions. By focusing on an underrepresented population, this study extends SDT-based MVPA research beyond Western contexts and underscores the importance of considering physical and behavioural correlates of motivational regulation when examining motivation for MVPA.

## Data Availability

The raw data supporting the conclusions of this article will be made available by the authors, without undue reservation.
